# The Application of Omics Technologies in Type II Diabetes Mellitus Research

**DOI:** 10.2174/0115733998362669250424162654

**Published:** 2025-06-03

**Authors:** Abdullah Al Sultan, Zahra Rattray, Nicholas J. W. Rattray

**Affiliations:** 1 Strathclyde Institute of Pharmacy and Biomedical Sciences, University of Strathclyde, 161 Cathedral Street, Glasgow, G4 0RE, UK;; 2 Faculty of Pharmacy, Kuwait University, Safat, 13110, Kuwait;; 3 NHS Lanarkshire, 50 Netherton St, Wishaw, ML2 0DP, UK

**Keywords:** Diabetes mellitus, type 2 diabetes, omics technologies, insulin resistance, disease biomarkers, toxico-omics

## Abstract

Diabetes mellitus represents a spectrum of chronic metabolic disorders characterized by elevated blood glucose levels (hyperglycemia), largely due to insulin deficiency or resistance. Type 2 diabetes mellitus (T2DM) is the most prevalent form, accounting for over 90% of diabetes cases globally. Its rising prevalence is a global concern, with projections indicating 783 million cases by 2045. T2DM leads to severe complications, including macrovascular diseases like cardiovascular events and microvascular issues such as retinopathy and neuropathy. Despite extensive research, the molecular mechanisms underlying T2DM are not fully understood. However, advancements in omics technologies, including genomics, proteomics, and metabolomics, have revolutionized diabetes research, notably in the following areas:

● The advent of single-cell sequencing has revealed cellular heterogeneity and dynamic changes during T2DM progression, paving the way for precision medicine approaches in diabetes research.

● Liquid chromatography-mass spectrometry (LC-MS)-based proteomics and metabolomics approaches have transformed T2DM research by enabling the discovery of early detection biomarkers, providing insights into key disease mechanisms and metabolic pathways, and facilitating the identification of novel therapeutic targets.

Despite challenges, integrating multi-omics data holds promise for unravelling the complex molecular networks involved in T2DM. This review explores recent advancements in omics research, its impact on T2DM, and future directions in the field.

## 1 INTRODUCTION

The term ‘diabetes mellitus’ is used to represent a spectrum of chronic, progressive metabolic disorders chiefly characterised by elevated levels of blood glucose (hyperglycaemia) [1, 2]. Diabetes mellitus is complex in aetiology and incompletely understood; however, the consensus is that a relative or absolute deficiency in insulin secretion, accompanied by varying degrees of peripheral resistance to insulin actions, a significant contributor to the imbalance in glucose homeostasis that manifests in the disease’s pathology [1-3]. The most common aetiologic classifications of diabetes include type 1 diabetes mellitus (T1DM), type 2 diabetes mellitus (T2DM), and gestational diabetes mellitus, with the vast majority of cases diagnosed as T2DM [1, 4].

### 1.1 Epidemiology of T2DM

T2DM is the most prevalent form of diabetes mellitus, accounting for more than 90% of all cases in adults [1, 4]. The prevalence of T2DM is increasing at a tremendous rate and is thus a pandemic concern [1, 4]. As of 2021, according to the International Diabetes Federation (IDF), roughly 537 million adults have been diagnosed with diabetes globally, and this number is projected to reach 783 million adults by 2045 [4]. In the US, in 2022, the Centers for Disease Control and Prevention (CDC) reported that diabetes mellitus affects more than 37 million Americans (1 in 10), the considerable majority of whom were diagnosed with T2DM (90-95%) [5]. Furthermore, in 2021, in the Middle East and North Africa region, the prevalence of adult diabetes was found to be 16.2% (73 million adults aged between 20 and 79), reflecting the highest estimated prevalence compared to other IDF regions [6]. Additionally, the region is estimated to experience the second-highest increase (86%) in the total number of diabetic subjects to 136 million by 2045 [6]. In the UK, in 2019, more than 4.9 million individuals were diagnosed with diabetes [7]. A National Health Service (NHS) report revealed that one in 15 people in the UK has diabetes, including one million people who have T2DM but have been underdiagnosed [7, 8]. Since 1996, the prevalence of diabetes in the UK has more than doubled, and if this trend continues, it is projected that more than 5.5 million people will have diabetes by 2030 [7, 8]. Most cases in the UK were diagnosed with T2DM, and the same NHS report (2019) indicated that 13.6 million people are at high risk of developing T2DM [7, 8].

### 1.2 Health and Economic Burden of T2DM

Patients with T2DM are at high risk of developing severe long-term complications, including macrovascular and microvascular complications (Fig. **1**) [1]. Studies have shown that patients with chronic metabolic imbalances have a two-to-six times higher risk of developing macrovascular complications, including ischemic heart disease, cardiovascular disease (CVD), and peripheral vascular diseases, compared to the general population [1]. Studies have reported that diabetic patients’ risk of developing myocardial infarction (MI) is equivalent to those who are non-diabetic with a previous history of MI [9]. Similar to coronary artery disease, diabetes is an independent risk factor for stroke and cerebrovascular diseases [9]. Diabetic patients have an increased risk of 150-400% for stroke compared to the general population [9]. Furthermore, the risk of vascular dementia and stroke-related deaths is elevated in diabetic patients [9]. Heart failure (HF), which is another leading cause of morbidity and mortality from CVD, has also been associated with diabetes mellitus [1, 9]. Recent studies have shown a twofold increase in HF-related hospitalisation among patients with diabetes compared to those without diabetes [1, 9].

Microvascular complications refer to diseases of the small blood vessels associated with the thickening of the basement membranes, which include diabetic retinopathy, diabetic nephropathy, and diabetic neuropathy. Chronic kidney disease (CKD) attributed to diabetes affects 20-40% of diabetic patients [10]. It typically presents at the time of T2DM diagnosis and is likely to progress to end-stage renal disease, which requires dialysis or kidney transplantation [1]. In fact, CKD attributed to diabetes is the leading cause of end-stage renal disease in the US [1, 10, 11]. Diabetic retinopathy is a progressive disease of the retinal microvasculature associated with persistent hyperglycaemia and other factors linked to diabetes, including hypertension [11]. The risk of developing diabetic retinopathy is strongly related to the duration of diabetes and the level of diabetic control [11]. Diabetic retinopathy is attributed to 4.8% of the 37 million cases of blindness worldwide and is considered the leading cause of blindness in patients aged 15-64 [10]. Diabetic neuropathy refers to a heterogeneous group of disorders characterised by nerve dysfunction that results in the loss of sensation, ulceration, and ultimately amputation [10]. Approximately half of all diabetic patients develop diabetic neuropathy within 25 years of disease onset [10]. Similar to other microvascular complications, the risk of developing diabetic neuropathy is proportional to the duration and level of glycaemic control [10]. Depending on the clinical manifestations and the site at which the peripheral nervous system is affected, diabetic neuropathy may manifest in several ways, including distal symmetric polyneuropathy and autonomic neuropathy [11]. Diabetic polyneuropathy is the most prevalent form of neuropathy in developed countries [12]. According to a large community-based study in the UK, the prevalence of clinical neuropathy among 15,692 diabetic patients was 49% [12]. This high prevalence rate indicates substantial morbidity consequences, including diabetic foot ulceration, which carries a risk of progressing to limb amputation [12]. In fact, more than 80% of amputations following foot ulceration or injuries are due to diabetic neuropathy [10]. A total of 40-60 million diabetic patients are affected by diabetic foot and lower limb complications globally [10].

Hence, diabetes mellitus is a global pandemic that poses a serious health and economic burden to all health care systems. The direct and indirect economic costs and related complications have a significant impact on national economies. In 2021, 6.7 million deaths were caused secondary to diabetes [4]. According to the American Diabetes Association's 2023 guidelines, macrovascular complications account for almost half of all diabetes-related cases of mortality and cost approximately $37.3Bn in CVD-related spending annually [1]. Furthermore, in the UK, the NHS spends approximately £ 10 Bn annually on diabetes [8]. The number of items prescribed for diabetes increased from 35.5 million in 2009 to 57.7 million in 2019 [8]. In 2021, the International Diabetes Federation stated that the total direct health expenditure for diabetes alone cost at least $ 966 Bn, labelling it the costliest chronic disease [5].

## 2 THE POTENTIAL ROLES THAT OMICS-BASED STUDIES CAN PLAY IN CURRENT T2DM RESEARCH

Despite the extensive research performed on the etiopathogenesis of T2DM, the molecular changes that contribute to disease onset and progression remain largely uncharacterised [13]. However, in the past decade, there has been a paradigm shift towards the utilisation of omics approaches to reshape the core biology of the disease and elucidate the mechanisms involved in T2DM development [13-19].

In recent years, a growing body of single-omics- and multi-omics-based studies has emerged, revolutionising T2DM research [13-19]. Together with advances in bioinformatics tools, the advent of high-throughput omics technologies has provided unprecedented opportunities to explore and redefine the T2DM research landscape [20-22]. Indeed, the emergence of functional genome-screening technologies (*e.g.*, whole genome and exome sequencing) and single-cell RNA-sequencing methods has enabled the deciphering of cellular gene expression heterogeneity and gene regulatory networks [20-22]. Furthermore, continuous innovation in mass spectrometry (*e.g.*, liquid chromatography-mass spectrometry (LC-MS)), particularly in terms of resolution and sensitivity, has led to the reliable identification and quantification of various cellular constituents, including proteins and metabolites [23, 24]. Advances in proteomic and metabolomic profiling techniques and protocols have therefore created new avenues for identifying novel T2DM biomarkers and therapeutic targets and thus providing insight into biological processes [23, 24].

Several examples support the transformative role of omics technologies. For instance, a quantitative gas chromatography-mass spectrometry (GC-MS) assay was conducted on sera samples from *db/db* mice and diabetic nephropathy patients to evaluate the potential of citric acid as a biomarker for early diabetic nephropathy diagnosis [25]. The results revealed cut-off citric acid concentrations of 29.24μg/mL, with a 95% confidence interval between 32.75 and 39.16μg/mL, in the diabetic nephropathy patients, and 16.74 and 22.57μg/mL in the normal controls [25]. Receiver operating characteristic curve (ROC) analysis revealed diagnostic accuracy levels exceeding 90% in both humans and *db/db* mice, suggesting that serum citric acid is a promising biomarker for detecting diabetic nephropathy [25].

Another study that highlights the transformative role of technology in advancing T2DM research was conducted by Alur *et al.*, who emphasised the utility of next-generation sequencing (NGS) in understanding T2DM [26]. Their analysis of the NGS dataset GSE81608 identified 927 differentially expressed genes (DEGs), comprising 461 upregulated and 466 downregulated genes, in T2DM patients. Pathway enrichment analysis revealed that the upregulated genes were notably associated with protein metabolism, with *NR1H3* and *NR1H2* playing key roles in regulating gene expression related to cholesterol transport and efflux. In contrast, the downregulated genes were significantly linked to metabolic processes, including the citric acid cycle and respiratory electron transport. Key hub genes, including *APP*, *MYH9*, *TCTN2*, *USP7*, *SYNPO*, *GRB2*, *HSP90AB1*, *UBC*, *HSPA5*, and *SQSTM1*, were pinpointed using protein-protein interaction networks and validated with ROC analysis, demonstrating their prognostic potential. These findings provide valuable insight into the genetic and molecular basis of T2DM, suggesting novel therapeutic targets [26].

Hence, compared to traditional bench science, which focuses on identifying and testing limited molecular targets, high-throughput omics-based studies - even studies conducted within a single omics field - often generate extensive data [27]. In turn, such data support the formulation of new research questions for a range of study types, from hypothesis- driven studies to data-driven scientific investigations [27].

Although it is clear that single omics-based studies and their outputs have value in T2DM research [13-19], T2DM is multifactorial in nature and involves a complex network of molecules from different molecular levels converging to manifest the disease phenotype [1-3]. Therefore, concerns about the design of omics-based studies have been addressed, and scholars have transitioned to integrating multi- omics datasets into their research [15-19]. It is evident from recent papers that the integration of multi-omics approaches is providing unparalleled opportunities to establish causality across different cellular function levels, thereby improving our comprehension of the interplay between molecular alterations and the phenotypic manifestation of T2DM [15-19].

This review aims to highlight recent advancements in omics-driven research that (i) elucidate the molecular mechanisms underlying disease onset and progression, (ii) improve T2DM risk predictions, and (iii) identify candidate T2DM biomarkers for diagnostic use. In this review, we have also addressed the contribution of the omics revolution to the identification of toxicological aspects of anti-diabetic agents. Finally, we have outlined the challenges faced in the omics era and speculated about the future use of omics in T2DM research.

### 2.1 Omics-driven Insights into the Multifactorial Nature of T2DM

Omics-based approaches have fuelled breakthroughs in various aspects of T2DM research, including disease pathogenesis [13-19]. For example, developments in genomic techniques in the last two decades have led to a broad array of genome studies being conducted, including linkage analyses, candidate gene studies, and large-scale association studies [28]. Such studies have helped define the potential role of genetics in T2DM [29, 30]. In one exciting development, approximately 70 T2DM-risk loci were identified shortly after the emergence of genome-wide association studies, accounting for 10% of overall T2DM susceptibility [29]. To date, large-scale, multiple-ancestry genetic studies have achieved tremendous progress, with over 500 novel risk loci identified [30]. Nevertheless, the genetic link to T2DM onset remains weak, as all the disease-associated-risk loci identified account for only 20% of T2DM heritability, augmenting the multifactorial nature of T2DM [30].

Interestingly, however, the complex interplay between genetics and T2DM incidence markedly accounts for T2DM risk variants primarily enriched in pancreatic islet enhancers [31]. Nevertheless, the gene targets of islet enhancers remain unknown [31]. Hence, Greenwald and colleagues performed an integrative analysis of chromatin loops in three islet samples using *in situ* Hi-C assays, Assay for Transposase-Accessible Chromatin with high-throughput sequencing (ATAC-Seq), and ChIP-seq to annotate islet enhancer variants and their target genes [32]. Through the genome-wide map generated *via* the combined analyses, putative target genes for thousands of distal islet enhancers and T2DM risk variants were identified [32]. Notably, the candidate target genes (Table **1**) of T2DM risk variants modulating islet enhancer activity, defined by chromatin looping and expression of a trait locus, including *CAMK1D*, *ABCB9*, *C2CD4B,* and *IGF2BP2*, were found to be particularly enriched in biological processes linked to protein transport and secretion pathways [32]. In parallel with the abovementioned findings, a reduction in islet enhancer activity and *IGF2BP2* expression was observed in T2DM-risk-linked SNPs [32]. These findings were supported by the results of an *in vitro* experiment in which conditional knockout of the *IGF2BP2* homolog Imp2 in mouse islets led to impairment in glucose-stimulated insulin secretion [32].

Recent progress in genome-wide association studies has significantly improved our ability to predict genetic predispositions to T2DM. One notable advancement is the polygenic risk score (PRS), which combines multiple genetic variants into a unified score, offering a more thorough assessment of an individual’s genetic susceptibility to the condition [33]. PRS is utilized to predict disease progression, optimize treatment selection, and identify subgroups of patients who may benefit from specific interventions [33]. The study in question aimed to develop and validate a trans-ancestry PRS using Bayesian polygenic modelling by integrating genome-wide association study data from populations of European, African, Hispanic/Latino, and East Asian ancestries [34]. The trans-ancestry PRS demonstrated a strong correlation with T2DM status across the different ancestral groups analysed. The top 2% of the PRS distribution demonstrated the ability to identify individuals with a 2.5-4.5 times greater risk of developing T2DM, which is comparable to the increased risk observed in first-degree relatives of individuals with T2DM. Additionally, a post hoc ancestry adjustment method was introduced to standardise the PRS across different populations, enhancing its utility as an equitable tool for clinical use [34].

In addition to the effect that genes have on T2DM pathology and risk prediction, the role that autoimmunity plays in T2DM pathogenesis has recently attracted interest due to new cases of T2DM arising following SARS-CoV-2 infection [35]. In this context, Wu *et al.* performed a single-cell analysis of the human pancreas using imaging mass cytometry [36]. They used a panel of 34 antibodies to discriminate between pancreatic islet cell types, exocrine cells, stromal components, and immune cells derived from 16 and 19 pancreata obtained from donors with T2DM and healthy donors, respectively [36]. The analysis revealed dramatic differences in the immune cell compositions, namely enrichment of HLA-DR-positive CD8 T cells and macrophages in the pancreata from the T2DM patients [36]. These findings generated a new avenue for understanding T2DM progression and revealed a potential role for immunotherapy in T2DM management [36]. In parallel with these findings, a gene regulatory network constructed by integrating three datasets (genomic, transcriptomic, and epigenomic) was utilised to gain a better understanding of the molecular mechanisms involved in T2DM pathogenesis [19]. Following data integration, downstream analysis was conducted using MSEA and wKDA to identify the key hub genes of T2DM and the implicated underlying pathways [19]. The MSEA enrichment analysis showed that the genes were particularly involved in glucose homeostasis and immune-related functions [19]. The top ten hub genes that resulted from the wKDA analysis were *PSMB9*, *COL1A1*, *COL4A1*, *HLA-DQB1*, *COL3A1*, *IRF7*, *COL5A1*, *CD74*, *HLA-DQA1,* and *HLA-DRB1*, revealing potentially novel biomarkers for use in T2DM research [19].

When exploring the pathophysiology of T2DM, it is necessary to consider insulin resistance (IR), which is a hallmark of T2DM [37]. IR is generally defined as a decreased biological response to the normal concentration of endogenous and/or exogenous insulin [37]. It may present for several years before the diagnosis of T2DM, with the capability to progress during the course of the disease [37]. The exact mechanism of IR is not entirely understood; nevertheless, numerous risk factors have been associated with its development [2]. Obesity, in the form of abdominal obesity and ectopic fat deposition (*e.g.*, in muscles and the liver), has been strongly implicated in the pathogenesis of IR [37]. The metabolic phenotype of obesity, chiefly characterised by the expandability of adipose tissue due to hyperplasia and/or hypertrophy, is a primary cause of excessive triglyceride deposition [38]. This, in turn, can lead to an alteration in adipose tissue function, a state termed adiposopathy [38]. During adiposopathy, the rate of lipolysis is high, leading to elevated levels of circulating free fatty acids [38]. It has been found that these high levels of circulating free fatty acids initiate an inflammatory state and cause IR [37, 39]. One explanation for the inflammatory state associated with obesity is that the secretory profile of adipocytokines (proteins secreted by adipose tissue) changes during adipocyte lipolysis [37, 39]. Numerous studies have reported increased production of adipocytokines, such as tumour necrosis factor (TNF)-α, or decreased release of protective adipocytokines, including adiponectin, in obese patients, which collectively are thought to mediate the effects of obesity in the pathogenesis of IR [37, 39]. Moreover, excess fat deposition in muscles or the liver, due to a relative deficiency of adipose tissue storage space in the usual fat storage sites, reduces insulin sensitivity and disrupts insulin signalling [37, 39, 40]. In addition to TNF-α, circulating free fatty acids—particularly high levels of palmitatic acid and arachidonic acid—may increase serine phosphorylation of IRS proteins, thereby reducing IRS1-associated PI3K activity, resulting in impairment in the regulation of GLUT4 and IR (Fig. **2**) [37, 39, 40].

In the context of the association between adiposopathy and T2DM incidence, it has been found that the prevalence of T2DM in Asian Indians is rapidly accelerating, primarily as a secondary condition to adiposopathy [41]. Surprisingly, there have been cases observed in individuals with a low body mass index, which have recently been attributed to a thin fat phenotype that is known to express higher cardiometabolic risk [41].

While the crucial role of visceral adipose tissue in T2DM pathogenesis has been defined, the impact of peripheral adipose tissue on T2DM is still unclear [41]. A whole transcriptomic analysis was therefore performed to ascertain the role that peripheral adipose tissue plays in T2DM [41]. A microarray study (30 T2DM patients *vs.* 30 nondiabetic individuals) of femoral subcutaneous adipose tissue (SAT) was initially conducted [41]. The DEGs and the perturbed pathways implicated by those genes were analysed using weighted gene co-expression network analysis and the KEGG database, respectively [41]. The top 20 DEGs revealed through transcriptome profiling were *HOXB3*, *RSPO3*, *HOXA5*, *GREM1*, *ORMDL1*, *C7*, *TRIM23*, *CLDN11*, *ABCA10*, *ETV5*, *TRIM2*, *TP53INP1*, *ST6GAL1*, *THBS2*, *ERAP1*, *OGT*, *RARRES1*, *CTDSPL* and *TBCC* [41]. The perturbed pathways were chiefly related to adipogenesis, oxidative stress, inflammation, and cross-talk between adipocytes/adipokines and immune cells (*e.g.*, the Toll-like receptor pathway) [41].

Saxena *et al.* continued their investigation of peripheral SAT by using an omics-based approach to examine its role in T2DM [42]. They performed whole transcriptome RNA-Seq of femoral subcutaneous adipose tissues to identify the key upstream regulatory factors implicated in disease pathogenesis [42]. The analysis revealed 2,752 DEGs significantly enriched in biological pathways linked to glucose and lipid metabolism; the findings aligned with those of previous microarray studies [42]. Furthermore, 65 miRNAs were differentially expressed, and eight of these (mir-133B, mir-210, mir-22, mir-25, mir-27A, mir-27B, mir-30E, and mir-503) were associated with T2DM, obesity, and inflammation (Table **2**) [42]. Based on the DEGs identified, a small network of 110 upstream regulatory proteins was constructed using eXpression2Kinases [42]. The network included transcription factors (TFs), protein kinases, and intermediate proteins [42]. Notably, all the TFs identified were implicated in adipogenesis [42]. Collectively, the findings generated by Saxena *et al.* foresee the molecular pathology of SAT and indicate that the impact of adiposopathy is not limited only to the visceral adipose tissue compartment [41, 42].

Interestingly, a recent study by Irgam *et al.* reported seven T2DM-associated highly significant variants in a cohort of 500 southern Asian Indians [43]. Using the AGENA MassARRAYiPLEX™ platform, the SNPs identified were s2241766-G (ADIPOQ), rs6494730-T (FEM1B), rs1799817-A, rs2059806-T (INSR), rs11745088-C (FST), rs9939609-A, and rs9940128-A (FTO) [43].

### 2.2 The Role of Omics-based Studies in the Profiling of Human Pancreatic Islet Cells

The emergence of single-cell technologies, including scRNA-seq, has marked a new era in revealing cellular heterogeneity and dynamics during disease progression [20-22]. As evident from recently published studies, scRNA-seq has proven its power in a wide range of applications, including novel cell population identification, cell-to-cell communication modelling, gene regulatory network prediction, and cell lineage tracing [44-46]. In a study involving pancreatic islets, single-cell transcriptome profiling of 638 islet cells derived from T2DM and nondiabetic subjects was performed to delineate cellular heterogeneity and identify the patterns of differences in islet cell-specific expression [44]. Using scRNA-seq, Lawlor *et al.* defined distinct gene expression signatures in T2DM and non-diabetic β, α, and δ cells that were due to cellular heterogeneity masked in previous molecular studies [44]. Furthermore, in quantitative terms, 248, 138, and 24 genes in the β, α, and δ cells, respectively, exhibited differential expression in T2DM individuals compared to nondiabetic individuals [44]. By employing the same concept of cellular heterogeneity and using scATAC-seq and deep-learning models, a research group identified cell-specific regulatory signatures (α, β, and δ) that underpin T2DM [45]. Interestingly, they also found T2DM-linked SNPs enriched in β-cell-specific and shared islet open chromatin [45]. Notably, the latter findings were not observed in α and δ cell-specific open chromatin [45]. In a recent meta-analysis of human islet scRNA-seq data obtained from six studies, the effect of T2DM on α- and β-cell transcriptomes was investigated [46]. The analysis included scRNA-seq results from 23 T2DM subjects and 47 healthy subjects, and the aims were (i) to identify the α- and β-cell subpopulations across the dataset and (ii) to perform a pathway analysis for the DEGs [46]. The pathway analysis findings suggested that the genes were involved in key energy metabolism, mitochondrial autophagy, and cellular stress processes that impact T2DM [46]. Additionally, upregulation of *NFE2L2*-regulated genes was noted in immature and senescent β cells, two subpopulations found in an increased proportion in T2DM subjects compared with healthy individuals [46].

Others have aimed to shape α-and β-cell behaviour using an epigenetic approach [47]. In this study, ATAC-seq was utilised to identify the open chromatin regions responsible for cell specificity and plasticity behaviours in human α and β cells [47]. The researchers successfully identified cell-type-specific open chromatin regions after integrating α- and β-cell ATAC-Seq data with mRNA-Seq data, and correlated the results with the cell-specific gene expression patterns [47]. Furthermore, the α- and β-cell ATAC-Seq peaks overlapped with recognised T2DM-linked SNPs and TF binding motifs, including those of *FOX* factors, *ISL1*, *MAFB*, *TFAP4,* and *SMAD2* [47].

### 2.3 Insights from Omics-based Studies into the Diagnosis of T2DM

An early diagnosis of T2DM is crucial for simultaneously maximising treatment strategies and minimising complications. However, traditional diagnostic methods used to determine plasma glucose and glycated haemoglobin (AC1) values require a considerable amount of time to complete and result in a relatively high occurrence of incorrect diagnoses [1]. Thus, in line with the increasing incidence of disease, the impetus for identifying T2DM diagnostic biomarkers continues to rise. Given the interest and success in using omics-based approaches to elucidate the pathogenic mechanisms at play in T2DM, it is not surprising that omics-based research is yielding promising diagnostic insights and that omics-based approaches are being explored to identify T2DM diagnostic biomarkers.

As previously reported, various genomic analyses, including genotype array studies, have been designed to determine the genetic risk factors for T2DM [28-30]. Ultimately, such work aims to identify promising diagnostic candidates suitable for disease screening, including SNPs in the *CDKLA1* [48], *KCNQ1* [49, 50], and *CDK2A/B* [51, 52] genes. The use of transcriptomics tools to landscape the coding (messenger RNA [mRNA]) and non-coding (microRNA [miRNA]) RNA profiles has also shown interesting diagnostic capabilities [53-55]. Numerous reports have highlighted the orchestral roles that miRNAs play in physiological and pathological processes [56]. The aberrant expression of these non-coding RNAs has been implicated in T2DM pathogenesis, predominantly in IR; consequently, this suggests that miRNAs might be novel biomarker candidates for T2DM diagnosis and that tracking changes in miRNA expression is warranted [56]. In combined analyses of quantitative reverse transcriptase polymerase chain reaction (qRT-PCR) and miRNA sequencing data, differential expression of multiple miRNAs, including miR-7, miR-101, miR-128, and miR-802, in plasma was associated with the onset of T2DM [57]. Furthermore, mRNA microarray analysis of saliva samples obtained from T2DM subjects revealed changes in the expression of various mRNAs, including Kirsten rat sarcoma 2 viral oncogene homolog, spermidine/spermine N1-acetyltransferase 1, and epidermal growth factor receptor, indicating that these are potential non-invasive diagnostic biomarkers for T2DM (Table **3**) [58].

In parallel with the recent implication of autoimmunity in T2DM pathogenesis, proteomics studies have been conducted to identify autoantibodies that signify the presence of the disease [59, 60]. For example, serum from T2DM patients was subjected to MS, and fragments of complement C3f and kininogen 1 isoform 1 precursor were found in association with T2DM onset [59]. In addition, another study that utilized protein microarrays and the Luminex system found that there were elevated levels of anti-PPAR2G2 and anti-UBE2M autoantibodies in the serum of T2DM patients compared to healthy subjects [60].

Further downstream of the genomic and proteomic changes associated with T2DM are metabolic changes related to the disease. There is clear evidence of metabolic perturbation in T2DM, and this provides the opportunity to use metabolomic methods to identify candidate biomarkers for disease detection. MS is widely used in metabolite detection, and several potential biomarkers have subsequently been revealed through the use of MS. For example, one study identified 1,5-anhydroglucitol (1,5-AG) as a diagnostic biomarker for T2DM using MS-based approaches [61].

Given that gut microbiome dysbiosis is associated with T2DM pathogenesis, the application of metagenomics techniques is proving to be beneficial in the quantitative measurement of microbial communities. Studies using 16S rRNA sequencing and shotgun metagenomics have found that there are changes in gut microbiota levels. Of significant note, *Desulfovibrio* and *Bacteroidetes caccae* have been implicated in T2DM [62] and therefore display potential as diagnostic biomarkers of disease.

Collectively, the findings described above provide a glimpse of the capabilities of omics technologies in biomarker identification. They also reveal that there are alternative avenues for the development of T2DM diagnostic tools and methods based on newly identified biomarkers.

### 2.4 Further Contributions of Omics-based Studies to Biomarker Discovery

Since the pathology of T2DM antedates the disease onset, numerous omics-based approaches encompassing epigenomics, metabolomics, and its subfield lipidomics have been adopted to identify biomarkers for early disease detection [63-68].

To investigate the role of DNA methylation patterns in T2DM pathomechanisms, several epigenome-wide association studies (EWASs) have been conducted, revealing potential DNA methylation markers associated with T2DM. For example, a recent systematic review analysed 15 EWASs [63]. The majority of the studies had a cross-sectional design, were blood-based, and were conducted with different ethnic groups [63]. In short, the analysis revealed 52 CpGs in T2DM subjects’ blood samples, five of which (*ABCG1*, *LOXL2*, *TXNIP*, *SLC1A5,* and *SREBF1*) were found to be significantly associated with T2DM and thereafter labelled as potential disease biomarkers [63].

Alongside epigenomics, metabolomics, as reflected by the techniques involved (*i.e.*, nuclear magnetic resonance spectroscopy and mass spectrometry), provides an extensive and simultaneous analysis of the large set of endogenous metabolites present in a biological sample [24]. Monitoring changes in the most downstream type of omics data (the metabolite content) during disease development enables the detection of biological markers and other applications of metabolomics tools. For example, chemical isotope labeling LC-MS was used to analyse serum samples from healthy normal weight subjects (n = 30), obese subjects (n = 26), and subjects newly diagnosed with T2DM (n = 16) and accordingly profile candidate biomarkers associated with either T2DM or IR [64]. Metabolic fingerprinting analysis revealed 42 perturbed metabolites correlated with T2DM, including amino acids, amino acid metabolites, and dipeptides [64]. Furthermore, numerous metabolites were identified as potential biomarkers of IR, including amino acids (asparagine, glutamine, and histidine), Met sulfoxide, 2-methyl-3-hydroxy-5-formylpyridine-4-carboxylate, serotonin, l-2-amino-3-oxobutanoic acid, and 4,6-dihydroxyquinoline [64]. In another metabolomics analysis performed on urine samples from 1,424 adults, the role of the urine metabolome in T2DM risk prediction was investigated [65]. Among the 62 preliminary annotated metabolites identified, three were associated with T2DM prevalence, including 3-hydroxyundecanoyl-carnitine, which was subsequently selected as a promising biomarker (Fig. **3**) [65].

Another study used a novel approach that combined metabolomics and imaging [66]. Metabolic profiling of SAT and plasma was performed in 42 individuals (T2DM, prediabetic, and healthy individuals) who underwent whole-body fluorine-18 fluorodeoxyglucose (18F-FDG) positron emission tomography (PET)/magnetic resonance imaging (18F-FDG PET/MRI) [66]. The primary objective was to identify any associations between the annotated metabolites and certain tissue-specific characteristics (volume, fat fraction, and insulin sensitivity [Ki for 18F-FDG]) of various tissues, namely the brain, heart, liver, visceral adipose tissue (VAT), and SAT [66]. In the context of plasma metabolome fingerprinting, positive and negative associations were noted between the hepatic fat content and tyrosine and lysoPC(P-16:0), respectively [66]. The insulin sensitivity of the VAT and SAT was found to be positively associated with several lysophospholipids and negatively associated with branched-chain amino acids [66]. For the SAT metabolome, the insulin sensitivity of the VAT was positively associated with non-esterified fatty acids [66]. The latter findings were consistent with hepatic insulin sensitivity [66]. However, inverse associations were observed between VAT insulin sensitivity and bile acids and carnitines [66].

Dyslipidemia is another hallmark of T2DM, and hence tracking the molecular changes in lipids potentially represents a promising model for screening and tracking T2DM progression. Recently, lipidomic profiling of plasma samples from 250 T2DM and 639 control participants showed that elevated levels of lysophosphatidylcholines and lysophosphatidylethanolamines (lysophospholipids [LPs]), phosphatidylcholine-plasmalogens (PC-PLs), sphingomyelins (SMs) and cholesterol esters (CEs) were inversely correlated with T2DM risk (multivariable-adjusted *p* for linear trend ≤0.001 for all), while higher levels of triacylglycerols (TAGs), diacylglycerols (DAGs) and phosphatidylethanolamines (PEs) were predominantly associated with a higher risk of T2DM (multivariable-adjusted *p* for linear trend <0.001 for all) [67]. In agreement with the latter findings, another lipidomic study performed with 277 plasma lipid samples showed lower levels of PC-PLs and elevated levels of TAGs and DAGs measured using UPLC-QToF-MS were correlated with a higher risk of developing T2DM [68].

### 2.5 The Role of Omics-based Approaches in Elucidating the Toxicity Mechanisms of Antidiabetic Agents

Over the past decades, the therapeutic management of T2DM has undergone major revisions due to advancements in evidence-based T2DM care [69, 70]. The ultimate aim of T2DM management is to maintain a level of glycaemic control that aids the prevention or delay of the onset and progression of T2DM-related complications [70]. The cornerstones of T2DM management include lifestyle interventions, concomitant pharmacological treatment, and self-monitoring of blood glucose [71]. Owing to the progressive nature of T2DM, a stepwise therapeutic approach is predominantly adopted in clinical practice, and diet regulation and exercise are typically recommended in the first instance [1, 70, 71]. However, if blood glucose levels remain high and inadequately controlled even with the employment of these measures, the administration of antidiabetic medications is warranted [1, 70, 71]. Currently, several medications are available for T2DM management, and they generally belong to the following major pharmacological classes: biguanides, sulfonylureas, meglitinides, thiazolidinediones (TZDs), dipeptidyl peptidase 4 inhibitors (DPP-4 inhibitors or gliptins), sodium-glucose cotransporter 2 (SGLT2) inhibitors, α-glucosidase inhibitors and glucagon-like peptide-1 receptor agonists (GLP-1 agonists) [71]. Antidiabetic medications lower the glucose level *via* one or more of the following mechanisms: (i) increase insulin availability by promoting insulin secretion from pancreatic β-cells, (ii) improve glucose uptake in peripheral tissues, (iii) delay the absorption of carbohydrates, (iv) increase satiety and regulate gastric emptying, (v) induce glucosuria, (vi) decrease hepatic glucose production, and/or (vii) suppress glucagon production from pancreatic α-cells [1, 70, 71]. The key data of the current pharmacological classes used for T2DM management are presented in Table **4**.

Despite the broad range of clinical benefits achieved through the design and development of antidiabetic drugs since the early 2000s, several drawbacks that may limit their use still exist. The most critical limitations are high cost and toxicity [1, 70]. Notably, the ADA 2023 classifies GLP-1 agonists, DPP-4 inhibitors, SGLT-2 inhibitors, and insulin analogues as high-cost medications [1]. Given that T2DM is progressive in nature, most patients need combination therapy that involves the use of two or more antidiabetic agents—often the high-cost medications mentioned above—to increase the chance of positive glycaemic, metabolic, and CVD outcomes (*e.g.*, GLP-1 agonists). This creates a substantial economic burden on patients and healthcare systems. Another concern that is corollary to the use of antidiabetic agents is the range of adverse effects. Recently, the list of pharmacological classes used for T2DM management has been expanded. As each class of compound has a unique mechanism of action, diverse toxicological profiles could consequently be induced by the various combinations of these antidiabetic drugs that typically require follow-up assessment and treatment. Clearly, with the unprecedented rise in the global prevalence of T2DM and its complications, there is an urgent need to optimise the safety profiles of individual antidiabetic agents and T2DM treatment protocols.

The principal objective in managing T2DM and its related complications is to maintain adequate glycaemic control, and whether this objective is achieved is primarily reliant on medication adherence [72]. Approximately 45% of T2DM patients fail to achieve their target HbA1c control level (<7%), and this is chiefly due to poor medication adherence [72]. Non-adherence is a serious health concern that is subsequently associated with higher incidences of T2DM-related complications, higher fatality rates, and increasing economic burdens on healthcare systems [72]. For example, in a study conducted in the UK, among 15,984 participants treated with antidiabetic agents, poor medication adherence was reported and independently associated with a significant 1.6-fold increase in all-cause deaths [72]. Regarding the reasons for non-adherence, multiple factors are associated with antidiabetic agent non-adherence, and one of the significant determinants of poor adherence is the occurrence of adverse effects [72]. Unfortunately, the majority of the adverse effects associated with the antidiabetic agents listed in Table **4** are poorly characterised. In fact, the pathomechanisms underlying these adverse effects are still poorly defined, and there is a dearth of mechanistic studies addressing these issues.

To date, conventional toxicological methods have typically been used to investigate the adverse effects of T2DM medications, mainly by observing toxicological endpoints. Despite the crucial findings that have emerged from the adoption of these methods, comprehensive descriptions of the biological pathways that mediate the toxic effects of the medications are still lacking. Thus, innovative methodological approaches are warranted to generate both comprehensive and targeted mechanistic data capable of filling existing data gaps and ultimately improving our understanding of drug toxicity. In recent decades, omics-based approaches, termed toxico-omics, have progressively become established as powerful tools in regulatory toxicology [73]. Moreover, the integration of toxico-omics and bioinformatics data modelling has caused a paradigm shift in the scope of toxicity-related investigations [21, 74]. This has efficaciously shifted the nature of experiments from observation-based studies to mechanism- and target-based analyses of the effect of specific xenobiotics on human biology [74]. As yet, various toxico-omics studies have been devoted to revealing the mode of toxicity of various drugs [73]. Importantly, these studies have successively reported the discovery of toxicity biomarkers while gaining a better understanding of the underpinnings of toxicity pathways [74-77].

In the studies that have investigated the toxic effects of antidiabetic agents and the inter-individual heterogeneity in the drug response among T2DM patients, the use of toxicogenomic methods is relatively common [78]. For example, a recent systematic review analysed 18 pharmacogenomic studies that involved a total of 10,383 T2DM patients using oral antidiabetic agents [78]. The main aim was to determine whether there is any association between genotype and adverse effects induced by oral antidiabetic agents [78]. Various SNPs in *SLC22A1*, *SLC29A4,* and *SLC6A4*, which are genes that encode drug transporters and metabolising enzymes, were found to be associated with GI intolerance attributed to metformin use [78, 79]. These adverse effects were shown to worsen with concomitant treatment with transporter-inhibiting drugs [78]. Regarding sulfonylureas, *CYP2C9* genetic variants were associated with a higher risk of sulfonylurea-induced hypoglycaemia [78, 80]. Furthermore, the CYP2C8*3 variant was associated with less weight gain in cases of rosiglitazone treatment [78, 81]. The C allele of rs6123045, an intronic SNP in *NFATC2*, was markedly associated with a higher rate of rosiglitazone-induced oedema [78, 81]. Of note, the interpretation of adverse drug reactions (ADRs) should also consider interethnic variations and their influence on susceptibility to ADRs and drug response. Ethnic diversity has long been recognized as a critical determinant of the genetics underlying variability in drug responses [82]. It is widely acknowledged that allele frequencies exhibit significant interethnic disparities [78, 82]. For instance, the loss-of-function variant CYP2C9*3 exhibits notable differences in frequency: ~6.7% in Europeans, ~1.3% in Africans, and ~1.2% in South Asians [78, 82]. These disparities highlight the need for personalised medicine and underscore the importance of integrating multi-omics approaches tailored to cohort-specific populations to advance precision medicine in diabetes. Such strategies can help optimise medication dosages to suit specific populations, ensuring both efficacy and safety.

Currently, no other omics-based studies have been conducted and reported to assess the effect of genetic polymorphisms on the incidence of adverse effects associated with other antidiabetic agents or to elucidate the mechanism of action of such adverse effects. Therefore, further toxicological studies are warranted to fill the existing knowledge gaps and support the design and development of preventive and therapeutic strategies to improve patient safety and, ultimately, medication adherence.

Apart from the contribution of omics-based approaches to elucidating the mechanisms of drug action and toxicity, the integration of multi-omics has shown success in repurposing drugs for T2DM research [83-85]. It is widely acknowledged that drug development is expensive and time-consuming. In contrast, drug repurposing based on omics data can be introduced into clinical practice quickly and at a reduced cost, which can help overcome limitations in drug design. In this context, a study was conducted to repurpose marketed drugs and clinical candidates for new diabetes treatments by analysing clinical ‘omics’ data, including genome-wide association studies, proteomics, and metabolomics [83]. The researchers identified 992 proteins as potential antidiabetic targets and retrieved drug information from the Therapeutic Target Database. Of these, 35 druggable proteins were selected, with 12 targets (linked to 58 drugs) showing potential for diabetes treatment. Using CMap to compare gene expression patterns, the study identified nine drugs with potential antidiabetic effects. Among these, four (diflunisal, nabumetone, niflumic acid, and valdecoxib) that target COX2 were repurposed for T1DM, while two drugs (phenoxybenzamine and idazoxan) that target ADRA2A showed promise for T2DM [83].

## 3 DISCUSSION

To tackle the existing healthcare burden of T2DM, researchers are now focusing their efforts primarily on omics-based approaches. This review summarises the major breakthroughs achieved in T2DM research *via* omics-based studies. Notably, it is evident that the majority of the studies performed in this area have adopted single-omics-based approaches. Despite the crucial findings that have emerged from these studies, several concerns must be addressed.

First, single omics-derived data are mainly associative and lack the resolving power required to establish causality between observed molecular perturbations and phenotypic manifestations. Furthermore, T2DM results from a complex heterogeneous interplay between genetic and environmental factors, and hence investigating the role that genetics plays in disease pathology without considering environmental influences largely confounds the reported findings. Similarly, within the context of single-cell analyses, delineating the complexity of a cell population requires the integration of multiple datasets derived from experiments conducted at different molecular levels.

Second, despite the fruitful progress made within individual omics strands, differences in the sensitivity of the tools used in different studies and the lack of harmonisation among the protocols adopted for sample preparation and data acquisition, processing, and analysis compromise the comparability of the findings across published studies. In turn, this impedes the incorporation of findings into risk assessment and decision-making. Therefore, the recent paradigm shift from single omics to multi-omics analyses is understandable.

Multi-omics, also known as multi-layered omics, is a biological research strategy that combines data from multiple “omics” layers [86]. This data integration approach provides deeper functional insights into biological systems and has wide-ranging applications in areas such as disease diagnosis, prognosis, and the identification of therapeutic targets (Fig. **4**) [86]. However, effectively integrating multi-omic data is a challenging process that requires the use of advanced and specialized techniques.

Various effective paradigms for integrating multi-omic data include consideration of subject-level information, analytical methods, and biological interpretation [86-88]. Broadly, subject-level integration in multi-omics is typically categorized into vertical and horizontal integration [87]. Vertical integration, or N-integration, involves incorporating different omics data from the same samples, utilizing concurrent observations across multiple functional levels [86-88]. In contrast, horizontal integration, or P-integration, combines studies conducted at the same molecular level on different subjects to increase the overall sample size [86-88]. Additionally, the multi-omics approach can be categorized into early and late integration, based on the stage at which the integration occurs [86, 87]. Early integration involves merging data from different omics platforms from the outset, before performing any classification or regression analysis, without considering the heterogeneity between the platforms [86, 87]. In contrast, late integration combines separate predictive models created for each omics layer, ignoring interactions between the different levels and the potential for synergy or antagonism [86]. Finally, the scope of biological interpretation is largely determined by whether integration is data-driven or knowledge-driven [87]. While a variety of data-driven and knowledge-driven approaches exist, their detailed descriptions, applications, advantages, and disadvantages are beyond the scope of this review and are comprehensively discussed elsewhere [86-90].

The primary aim of integrating multi-omics techniques is to fill the gaps that remain following the application of single-omics-based approaches. Several case studies have highlighted successful applications of multi-omics approaches in T2DM cases. In one study, researchers investigated the causes of gastrointestinal dysfunction in T2DM using *db/db* mice as a model [91]. A comprehensive multi-omics analysis encompassing the gastric sinus metabolome, transcriptome, and proteome revealed that T2DM mice exhibited significantly lower small intestine propulsion, gastric emptying rates, elevated blood glucose levels, and increased obesity compared to control mice. The study identified 297 genes, 350 proteins, and 1,001 metabolites that were differentially expressed between the two groups. Pathway analysis revealed that these molecules were involved in regulating arachidonic acid metabolism, glycerophospholipid metabolism, and vitamin digestion and absorption, providing valuable insights into the disease's pathomechanisms [91]. Using multi-omics, researchers revealed alterations in intestinal flora and plasma protein metabolic profiles in the Zucker diabetic fatty rat, a common model for T2DM [15]. Altered intestinal microbiota, including the genera *Phocea* and *Pseudoflavonifractor* and the species *Lactobacillus intestinalis*, along with differential protein and metabolite expression, effectively distinguished treated rats from control animals, providing valuable insights for T2DM screening and diagnosis. Pathway analysis of these molecules revealed key pathophysiological mechanisms of T2DM, focusing on oxidative stress, insulin resistance, and metabolic disorders. These data confirmed previous research on T2DM mechanisms and offered insights into overall molecular levels [15]. In an interesting recent study, the predictive power of combined omics biomarkers (genome, proteome, and metabolome) alongside that of clinical factors was evaluated to identify individuals at high risk of developing T2DM [92]. With a prospective cohort of 1,105 participants, the study compared prediction models that incorporated omics data with clinical biomarkers, such as variables from the Cambridge Diabetes Risk Score and HbA1c. The results showed that while proteomics data alone achieved the highest performance (C index = 0.82), combining omics features with clinical factors led to a modest improvement in predictive performance (C index = 0.87). The greatest benefit was observed in individuals with an HbA1c < 42 mmol/mol (the prediabetes threshold), where the use of PRSs significantly improved predictive power. Nevertheless, compared to those with prediabetes, individuals with high polygenic risk in this group had approximately half the overall risk of developing type 2 diabetes over 20 years, suggesting limited practical value for genetic screening in population-based prevention efforts [92].

It is worth mentioning here that there is still work to be done to improve the translation of omics-based findings into effective clinical practice. In addition, multi-omics-based studies represent a growing field of research, and there are real challenges and concerns associated with their design that should be thoughtfully considered. For example, when conducting a joint integration analysis, the differences in the maturity levels of the various omics strands are a significant constraint [87]. For instance, the massive differences in the scale and variance of the data obtained using metabolomics tools (a growing field) and genomics tools (the most mature field) in a simultaneous analysis can lead to the domination of one data type over the other [87]. This is also a concern when conducting a joint pathway analysis to reveal potential molecular mechanisms implicated in a disease pathology and a drug’s mode of action [87]. Such analyses usually rely on lists of genes, proteins, and metabolites annotated in a reference database (*e.g.*, the KEGG). Most human genes and proteins are well-annotated; however, only 22% of the annotated metabolites in the largest collection of metabolomics data (the HMDB) are mapped to biological processes [87]. This variation in reference annotations requires careful consideration during data interpretation [87]. Furthermore, variation in the study design adopted, analytical pipelines utilised, and strategies used for data integration (beyond the scope of this review) represent another challenge faced in the omics era. Hence, progressive efforts to optimise, harmonise, and standardise the protocols adopted in multi-omics data analyses and their interpretation are essential to ensure the translation of omics-driven insights into clinical outcomes. Another limitation is the complexity involved in data analysis and interpretation. Integrating data from various omics layers requires advanced bioinformatic tools and specialised expertise. Additionally, the high cost of conducting large-scale omics studies can restrict accessibility.

## CONCLUSION

Collectively, the information presented in this scoping review highlights the potential that exists in T2DM research in this era of omics. However, to ensure that the findings emanating from omics-based studies are efficiently translated into clinical outcomes, it is necessary to optimize and standardize study designs and develop tools that support the integration and interpretation of multiple omics-derived datasets. Addressing existing gaps in T2DM omics research is critical to this endeavor, as these gaps highlight key areas requiring further investigation. The underrepresentation of diverse populations restricts the generalizability of findings and hinders the discovery of population-specific biomarkers and therapeutic targets. Similarly, the limited exploration of sex-based differences in T2DM molecular pathways represents a missed opportunity for developing tailored treatments. The intricate interplay between epigenomic modifications and environmental factors, such as diet and lifestyle, remains insufficiently understood, impeding a comprehensive understanding of gene-environment interactions in T2DM. Moreover, translating omics discoveries into clinical applications is challenged by the persistent need for functional validation. Lastly, the limited integration of omics data with clinical and phenotypic information constrains advancements in precision medicine for T2DM. Addressing these challenges is essential to unlocking the full potential of omics research to enhance T2DM understanding and management.

Emerging trends in omics technologies, however, offer transformative potential to bridge these gaps and propel T2DM research forward. Recent progress in single-cell and spatial omics enables a remarkably detailed view of cellular heterogeneity, providing profound insights into sex-specific variations and the roles of diverse cell populations in T2DM pathogenesis [93, 94]. Single-cell omics delves into cellular heterogeneity, uncovering sex-specific differences in gene expression and metabolic pathways driving the disease, while spatial omics reveals the spatial organization of cells and their microenvironmental interactions, illuminating how these factors influence disease progression [93, 94]. In response to challenges in multi-omics integration, the rise of artificial intelligence (AI) and machine learning (ML) has provided indispensable tools for merging complex datasets with clinical and phenotypic information, uncovering latent patterns, and enabling the discovery of population-specific biomarkers [95, 96]. Furthermore, advancements in epigenetic editing and functional genomics, through technologies such as Clustered Regularly Interspaced Short Palindromic Repeats (CRISPR) and other genome-editing tools, including Transcription Activator-Like Effector Nucleases and zinc finger nucleases, are enabling researchers to move beyond correlation and establish definitive causal links between molecular features and T2DM phenotypes [97, 98]. Finally, innovative MS-based imaging techniques, such as Matrix-Assisted Laser Desorption/Ionization Mass Spectrometry Imaging (MALDI-MSI) and Desorption Electrospray Ionization Mass Spectrometry Imaging (DESI-MSI), have emerged as transformative tools, offering spatially resolved molecular mapping of key biomolecules in tissues like the pancreas and liver [99, 100]. These cutting-edge approaches collectively pave the way for addressing critical gaps, advancing our understanding of T2DM mechanisms, and unlocking new avenues for precision medicine.

To conclude, current and future omics technologies should be used to develop progressive toxico-omics studies and initiatives to awaken the dormant field of mechanistic toxicology in T2DM research and elucidate the cellular disturbances that occur during treatment with antidiabetic agents. Future omics research should also focus on integrating multi-omics approaches to advance precision medicine in diabetes. While the integration of various layers of omics datasets across different cohorts has demonstrated success in identifying reliable biomarkers for T2DM, significant benefits can be achieved by utilizing multi-omics measurements within the same individuals. For instance, case studies, including those using the UK Biobank, highlight the value of integrating genotyping and proteomic data through protein quantitative trait loci analyses to uncover the genetic regulators of protein expression and establish causal links between biomarkers and diseases using Mendelian randomization frameworks [33]. This comprehensive approach holds immense potential for tailoring personalized interventions and improving diabetes management.

## Figures and Tables

**Fig. (1):**
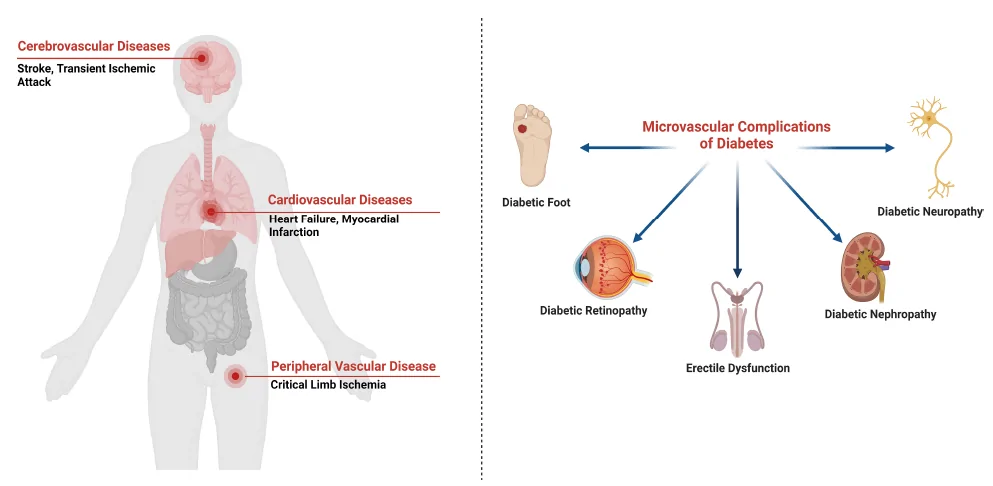
Macrovascular and microvascular complications of type II diabetes mellitus. Created with BioRender.com.

**Fig. (2):**
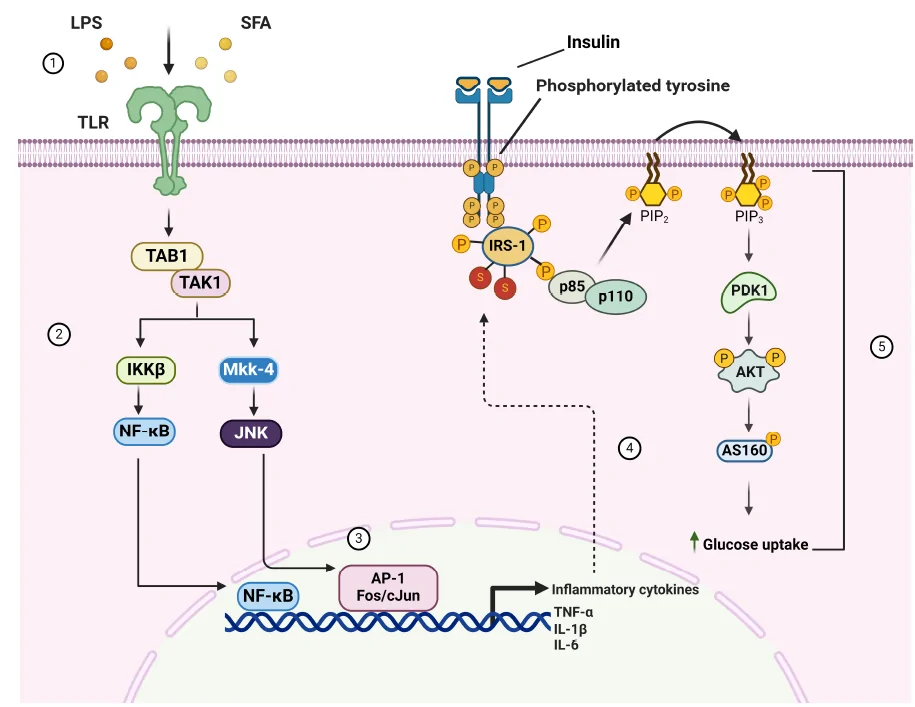
Fatty acid-induced insulin resistance. The activation of TLRs by their ligands (SFA or LPS) causes the assembly of TAK1 kinase with TAB1, TAK1-TAB1 complex (1), which promotes the activation of JNK and IKKβ (2). This subsequently activates transcriptional genes (*e.g.*, NF-κB and AP-1), which are responsible for upregulating the transcription of inflammatory genes (TNF-α, IL-1β, IL-6) (3). These inflammatory genes increase serine phosphorylation of IRS proteins (4), thereby reducing IRS1-associated PI3K activity, resulting in impairment of the regulation of GLUT4 and IR (5). LPS: lipopolysaccharides; SFA: saturated fatty acid; TLR: toll-like receptors; TAK1: transforming growth factor β-activated kinase 1; TAB1: transforming growth factor β-activated protein kinase 1-binding protein 1-β; NF-κB: nuclear factor kappa B; JNK: c-JUN amino-terminal kinase; IKKβ: inhibitor of nuclear factor kappa B kinase subunit β; Ap-1: activator protein 1; IRS: insulin receptor substrate; TNF-α; tumour necrosis factor α; IL-1β: interleukin 1β; IL-6: interleukin 6; Akt: protein kinase B; PDK1: phosphoinositide-dependent protein kinase 1; PIP2: phosphatidylinositol-4,5-biphosphate; PI3K: phosphatidylinositol kinase 3; PIP3: phosphatidylinositol-3,4,5-triphosphate; PKC: protein kinase C. Created with BioRender.com.

**Fig. (3):**
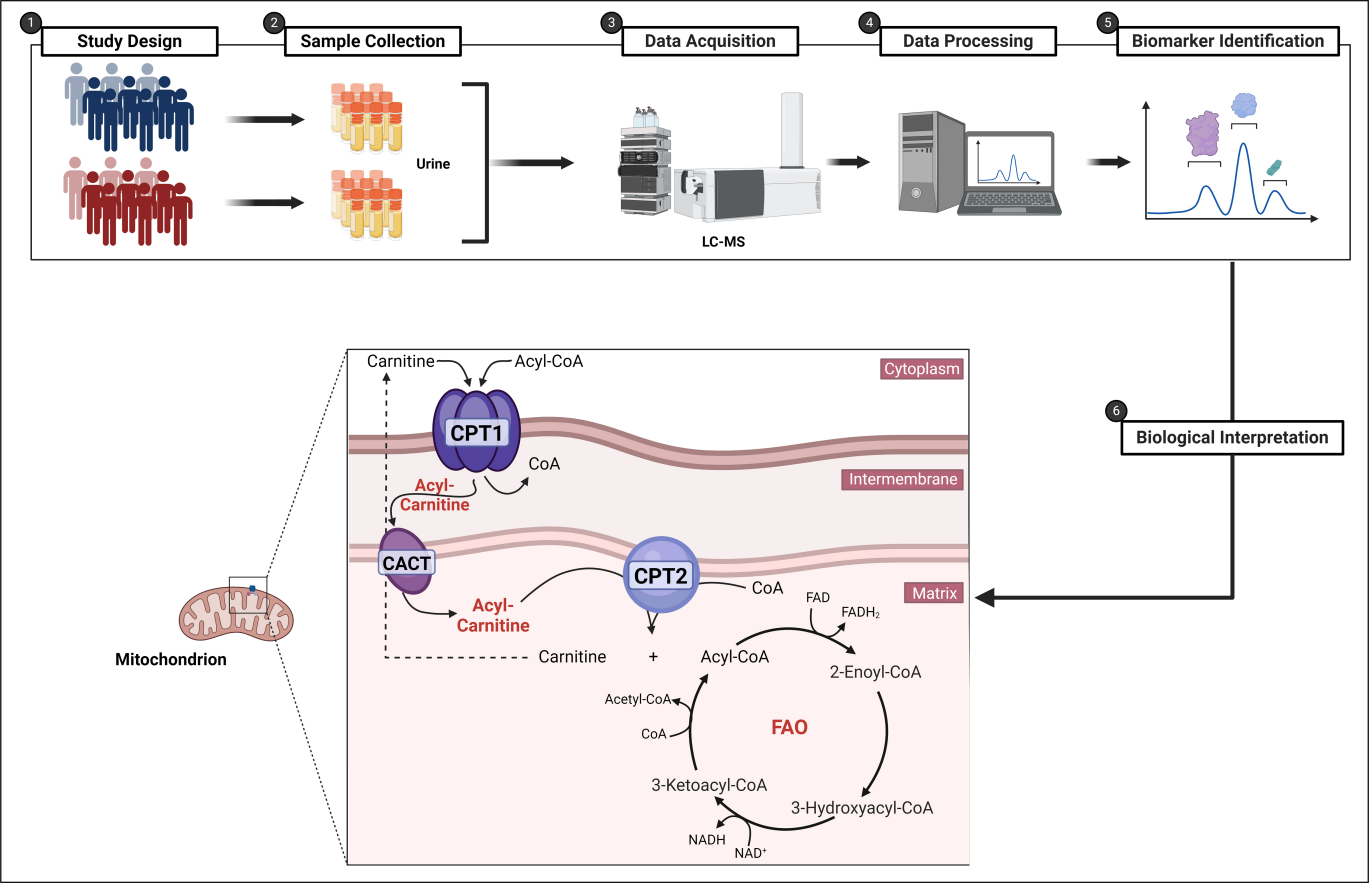
Graphical representation of non-targeted urine metabolomics analysis by Salihovic *et al.* to identify biomarkers associated with type 2 diabetes mellitus prevalence and incidence. (1, 2) non-targeted metabolomics was performed on urine samples from two community-based cohorts of over 1,400 Swedish adults using (3) ultra-performance liquid chromatography/mass spectrometry (UPLC-MS). Following data processing (4), three out of 62 annotated metabolites were found to be associated with prevalent T2D, including notably lower urine levels of 3-hydroxyundecanoyl-carnitine (5). The study suggests that 3-hydroxyundecanoyl-carnitine, a metabolite extensively involved in fatty acid metabolism (6), could serve as a biomarker candidate for T2DM. However, further analyses are needed to fully establish its role in T2DM risk prediction. Created with BioRender.com [65].

**Fig. (4):**
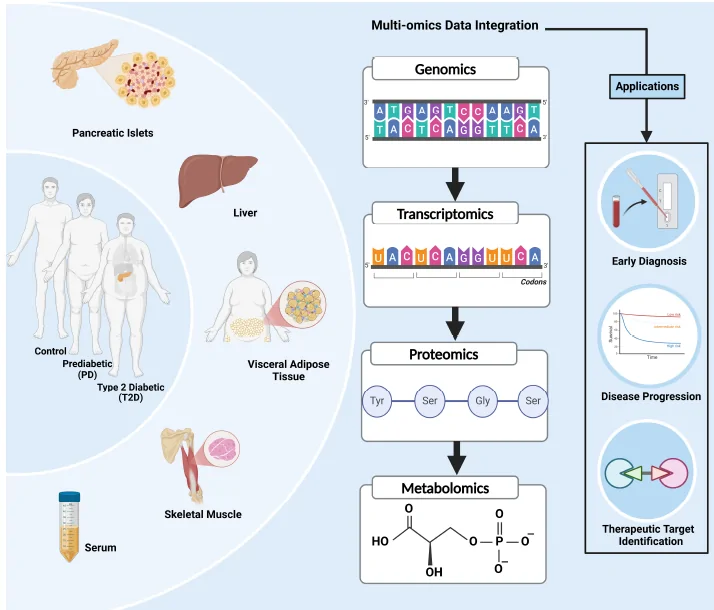
Applications of multi-omics in type 2 diabetes mellitus research. The integration of multi-omics analyses has provided valuable insights into T2DM research. Each omics layer—spanning the genome, transcriptome, proteome, and metabolome—captures increasing levels of biological complexity. This comprehensive data, reflecting deep phenotyping of individuals, holds the potential to refine disease diagnosis, prognosis, and the identification of therapeutic targets. Created with BioRender.com.

**Table 1:** Identified genes with eQTLs (corrected *p*<0.05) related to T2DM enhancer variants.

**Locus**	**Enhancer Variant**	**eQTL Gene(s)**	**eQTL Gene(s) Names**	**Shared eQTL**
10p13	rs11257655	CAMK1D	Calcium/Calmodulin Dependent Protein Kinase ID	Y
8p11	rs508419	NKX6-3	NK6 Homeobox 3	Y
12q24	rs1260294	ABCB9	ATP Binding Cassette Subfamily B Member 9	Y
3q27	rs7646518	IGF2BP2	Insulin Like Growth Factor 2 mRNA Binding Protein 2	Y
2q21	rs4954179	ACMSD	Aminocarboxymuconate Semialdehyde Decarboxylase	Y
		TMEM163	Transmembrane Protein 163	
15q22	rs17205526	C2CD4B	C_2_ Calcium Dependent Domain Containing 4B	Y
4q35	rs116401167	ACSL1	Acyl-CoA Synthetase Long Chain Family Member 1	Y
5q13	rs7732130	PDE8B	Phosphodiesterase 8B	Y

**Abbreviation:** eQTL: Expression quantitative trait loci.

**Note: ** [32].

**Table 2:** Differentially expressed miRNAs linked to T2DM and its associated complications.

**S. No.**	**microRNA**	**Fold Change**	**Significance (*p* < 0.05)**	**miRNA-Disease Associations in T2DM and Related Complications**				
				**T2DM**	**Diabetic Retinopathy**	**Diabetic Nephropathy**	**Obesity**	**Inflammation**
1	hsa-mir-133B	3.02	0.01495	✓	✓	-	-	✓
2	hsa-mir-210	7.56	0.03054	✓	-	-	✓	✓
3	hsa-mir-22	2.68	0.040103	✓	-	-	-	✓
4	hsa-mir-25	0.29	0.012915	-	-	✓	-	✓
5	hsa-mir-27A	7.68	0.003672	✓	✓	✓	✓	✓
6	hsa-mir-27B	0.34	9.97E−05	✓	✓	✓	✓	✓
7	hsa-mir-30E	0.07	0.000397	✓	-	✓	✓	-
8	hsa-mir-503	0.18	0.046336	✓	-	-	-	-

**Note: ** [42].

**Table 3:** Receiver operating characteristic curve evaluation of the identified salivary transcriptomic biomarkers.

**Gene symbol**	**Gene Name**	**AUC (95% CI)**	***p*-value**	**Sensitivity (%)**	**Specificity (%)**
KRAS	Kirsten Rat Sarcoma Viral Oncogene Homolog	0.728	0.035	84.6	69.2
SLC13A2	Solute Carrier Family 13 Member 2	0.769	0.032	69.2	84.6
SAT1	Spermidine/Spermine N1-Acetyltransferase 1	0.799	0.006	69.2	76.9
TMEM72	Transmembrane Protein 72	0.692	0.031	46.2	100
PSMB2	Proteasome Subunit Beta Type-2	0.728	0.039	69.2	76.9
EGFR	Epidermal Growth Factor Receptor	0.746	0.034	100	46.2

**Abbreviation:** AUC: Area under curve.

**Note: ** [58].

**Table 4:** Key data of current pharmacological classes used for T2DM.

**Pharmacological Class**	**Mechanism of Action**	**HbA1c Reduction (%)**	**Additional Benefits**		**Cost**	**Safety Concerns**
			**CV Effects**	**Renal Effects**		
**Biguanides**	-Decrease hepatic gluconeogenesis and increase insulin sensitivity	1-2%	Potential ASCVD benefits	Neutral	Low	-GI (diarrhoea, abdominal pain) -Potential for B12 deficiency -Lactic acidosis
**TZDs**	-Increase insulin sensitivity	0.5-1.4%	Potential ASCVD benefits (Pioglitazone)	Neutral	Neutral	-Weight gain -Fluid retention -Congestive heart failure -Bone fractures
**Sulfonylureas**	-Enhance insulin secretion	1-2%	Neutral		Low	-Hypoglycaemia -Weight gain
**Meglitinides**	-Enhance insulin secretion	0.5-1.5%	Neutral		Low	-Hypoglycaemia -Weight gain
**GLP-1 agonists**	-Increase satiety, regulate gastric emptying, enhance insulin secretion and decrease glucagon release	0.5-1.5%	ASCVD benefits	Reduce diabetic kidney disease progression (Liraglutide)	High	-GI (diarrhoea, abdominal pain) -Injection site reactions
**DPP-4 inhibitors**	-Increase endogenous GLP-1 levels	0.5-0.8%	Neutral		High	-Increased risk of infection -Headache -Arthralgia
**SGLT2 inhibitors**	-Induce glucosuria and prevent glucose reabsorption in the kidney’s proximal tubule	0.3-1%	Both ASCVD and HF benefits	Reduce diabetic kidney disease progression	High	-Genitourinary infection -Hypovolemia -Renal insufficiency -Hypotension -Diabetic ketoacidosis
**α-glucosidase inhibitors**	-Slow the absorption of carbohydrates	0.5-0.8%	Neutral		Mod.	-GI (flatulence, diarrhoea)

**Abbreviations:** TZDs: thiazolidinediones; DPP-4: dipeptidyl peptidase 4; SGLT2: sodium-glucose cotransporter 2; GLP-1 agonists: glucagon-like peptide-1.

**Note: ** [1, 70].

## LIST OF ABBREVIATIONS

T2DM: Type 2 Diabetes Mellitus
IDF: International Diabetes Federation
CDC: Centers for Disease Control and Prevention
NHS: National Health Service
CVD: Cardiovascular Disease
MI: Myocardial Infarction
HF: Heart Failure
CKD: Chronic Kidney Disease
